# The CD4^+^ T cell methylome contributes to a distinct CD4^+^ T cell transcriptional signature in *Mycobacterium bovis*-infected cattle

**DOI:** 10.1038/srep31014

**Published:** 2016-08-10

**Authors:** Rachael Doherty, Ronan Whiston, Paul Cormican, Emma K. Finlay, Christine Couldrey, Colm Brady, Cliona O’Farrelly, Kieran G. Meade

**Affiliations:** 1 Animal & Bioscience Research Department, Teagasc, Grange, Co. Meath, Ireland; 2 Comparative Immunology Group, School of Biochemistry and Immunology & School of Medicine, Trinity Biomedical Research Institute, Trinity College Dublin, Ireland; 3 Livestock Improvement Corporation, 605 Ruakura Rd, Newstead, Hamilton, New Zealand; 4 Central Veterinary Research Laboratories, Department of Agriculture, Food and the Marine, Longtown, Co. Kildare, Ireland.

## Abstract

We hypothesised that epigenetic regulation of CD4^+^ T lymphocytes contributes to a shift toward a dysfunctional T cell phenotype which may impact on their ability to clear mycobacterial infection. Combined RNA-seq transcriptomic profiling and Reduced Representation Bisulfite Sequencing identified 193 significantly differentially expressed genes and 760 differentially methylated regions (DMRs), between CD4^+^ T cells from *M. bovis* infected and healthy cattle. 196 DMRs were located within 10 kb of annotated genes, including GATA3 and RORC, both of which encode transcription factors that promote T_H_2 and T_H_17 T helper cell subsets respectively. Gene-specific DNA methylation and gene expression levels for the TNFRSF4 and Interferon-γ genes were significantly negatively correlated suggesting a regulatory relationship. Pathway analysis of DMRs identified enrichment of genes involved in the anti-proliferative TGF-β signaling pathway and *TGFB1* expression was significantly increased in peripheral blood leukocytes from TB-infected cattle. This first analysis of the bovine CD4^+^ T cell methylome suggests that DNA methylation directly contributes to a distinct gene expression signature in CD4^+^ T cells from cattle infected with *M. bovis*. Specific methylation changes proximal to key inflammatory gene loci may be critical to the emergence of a non-protective CD4^+^ T cell response during mycobacterial infection in cattle.

Bovine tuberculosis (BTB) is one of the most complex and persistent infections facing the agricultural industry^1^. An effective adaptive immune response is critical for host resistance to mycobacterial infection^2^ and CD4^+^ T cells are major drivers and regulators of this response^3^,^4^,^5^. As the predominant producers of the protective cytokine Interferon γ, T_H_1 CD4^+^ T cells are considered the key effector cell type in successful clearance of TB infection. In contrast, the development of clinical disease is associated with a shift toward an ineffective T_H_2/IL-4 mediated response suggesting CD4^+^ T cell dysfunction^6^. In humans, mycobacterial infection is associated with progressively dysfunctional T cell responses, which include impaired cytokine production and proliferative capacities^7^. Although possible reasons for the phenotypic switch include underlying genetics, infection load and T cell exhaustion, the precise drivers of the phenotypic and functional shift during BTB remain unclear^8^. Relevantly, recent research on *M. tuberculosis* has started to illuminate the mechanisms by which specific virulence proteins alter the cytokine milieu to induce a more favorable T cell phenotype for their survival^9^.

BTB infection in cattle induces significant changes in the numbers of innate (neutrophils and macrophages) and adaptive immune cell populations in circulation. These changes are reflected at a molecular level, with distinct differences in the immune profile of circulating cells reliably differentiating between infected and control groups on the basis of infection status^10^,^11^. In the same studies, widespread suppression of gene expression, particularly of innate immune genes, has been described in TB infected cattle. Follow on work analysing macrophage responses to *M. bovis in vitro* support these *in vivo* findings^12^ with enrichment of down-regulated genes in top-ranking canonical immune relevant pathways including *Leukocyte Signalling*^13^.

In this study, we hypothesised that *M. bovis* infection induces a specific CD4^+^ T cell functional phenotype that may impact on the ability of the host to clear mycobacterial infection. We further aimed to determine whether compromised CD4^+^ T cell phenotype and function in BTB infection is epigenetically regulated. As a predominant mechanism of epigenetic regulation, DNA methylation causes chemical changes in DNA without alterations in the underlying DNA sequence. These changes dictate cellular phenotypes via altered gene expression profiles^14^, and as a result can potentially determine the direction and potency of an immune response during mycobacterial infection.

Recent gene-specific analysis showed that bovine T cell cytokine production (IFNG and IL4) is regulated by DNA methylation^15^. We have also shown that epigenetic mechanisms contribute to the immune responsiveness of peripheral blood cells to the bacterial endotoxin, LPS^16^. While the recent assembly of the bovine genome, followed by technological developments in genomics has accelerated understanding of the pathogenesis of BTB^17^,^18^, the role of DNA methylation in regulating the CD4^+^ T-cell responses during mycobacterial infection in cattle has not been explored. It is likely that understanding epigenetics can offer new insights into the maladaptive functioning of the immune response during progressive mycobacterial infection^19^. Murine models of TB are showing how *M. tuberculosis* virulence proteins can manipulate the host immune response via methylation and repress genes involved in the first line of defence against mycobacteria^20^. Therefore, this study focused on identifying differences in the CD4^+^ T lymphocyte response induced during natural *M. bovis* infection, using RNA-seq gene expression analysis and sought to determine the regulatory contribution played by the CD4^+^ T lymphocyte DNA methylome.

## Results

### CD4 T lymphocyte numbers are similar in TB infected cattle but transcriptional profiles are different

Absolute lymphocyte numbers were similar between groups, although absolute polymorphonuclear neutrophil numbers were depleted in TB infected cattle, relative to healthy control (HC) cattle (Figure S1). To assess lymphocyte subpopulations, flow cytometry was used to identify the relative proportions of CD4^+^, CD8^+^ and γδ T lymphocytes in peripheral blood mononuclear cells (PBMCs) [Figure S2]. No significant difference in the proportions of CD4^+^ or CD8^+^ cells was detected in the TB infected animals. Although a significantly higher proportion of WC1^+^ γδ T lymphocytes was detected in the BTB group (Figure S2); these cells are not retained in the selected CD4^+^ population, and so are not considered further.

Magnetically-purified CD4^+^ T lymphocytes from both TB and HC (n = 5/group) were subsequently selected, RNA was extracted and sequenced on an Illumina HiSeq. Bioinformatic analysis (including stringent quality control filtering) of the resulting data was then performed to identify differentially expressed genes. An average of >73 million 75 bp paired-end reads were generated from each CD4^+^ T cell RNA sample and the percentage mapping efficiencies ranging from 59.7% to 70.5% - for mapping statistics see Table S1.

**Figure 1:**
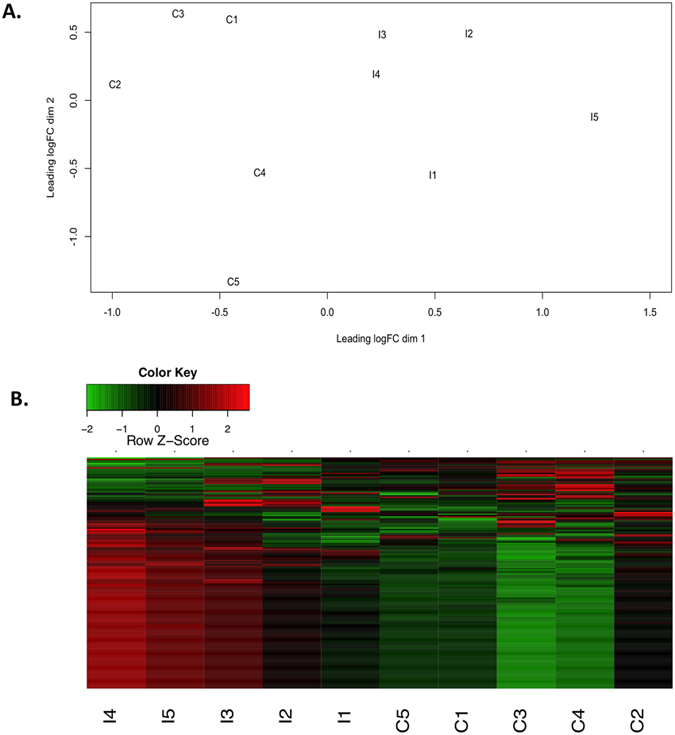
Gene expression profiles of CD4^+^ T lymphocytes from healthy controls and *M. bovis* infected cattle. The similarity in expression profiles between all animals (n = 10) was visualised using an MDS plot, produced with EdgeR (**A**). The read counts for individual genes were compared between all biological replicates. Control animals (C1–C5) were clustered seperately from infected animals (I1-15) on the basis of transcriptomic profiles. 95 genes that were significantly differentially expressed with an FDR <0.05 and log_2_FC +/−2 are shown (**B**). Upregulated gene expression is shown in red whereas downregulated expression is shown in green. The colour change represents z-scores that are calculated for each gene. Z-scores scales each gene to have a mean = 0 and standard deviation = 1.

A multi-dimensional scaling plot was created in order to visualise the similarity across samples, on the basis of a pairwise comparison of their CD4^+^ T lymphocyte transcriptomic profiles. Results showed that samples separated into two distinct groups on the first dimension (Fig. 1A). This clear partitioning of HC and TB infected samples indicates that a distinct gene expression profile exists in CD4^+^ T lymphocytes from *M. bovis* infected animals. Using an FDR cutoff of 0.05, a total of 193 genes were differentially expressed between both groups (Table S2) and 95 of these were found to have a log_2_ fold change >2. The range of log_2_ fold changes varied from −4.9 to 10.8, although 33 of the most highly differentially expressed genes remain uncharacterized in cattle. The majority (138) of differentially expressed genes were more highly expressed in the CD4^+^ T cells from *M. bovis* infected cattle, as can be seen from the heat map in Fig. 1B. Increased expression was detected, in the TB infected cattle, for Dual specificity protein phosphatase genes (*DUSP4* and *DUSP5*), the gene encoding alkaline phosphatase (*ALPL*), interferon induced protein 5 (*IFIT5*), interleukin 18 receptor accessory protein (*IL18RAP*), the lymphocyte chemokines *CCL4*, *CXCL2* as well as *IL8* and receptor gene *CXCR4* (for full list see Table S2).

**Figure 2:**
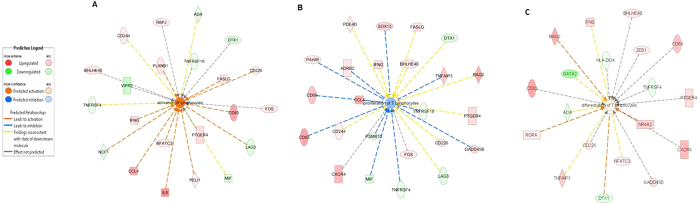
Pathway analysis identified a significant enrichment of genes involved in the process of T cell activation, proliferation and differentiation. Included are differentially expressed genes (FDR **<**0.1) known to be involved in the process of T lymphocyte activation (**A**), proliferation (**B**) and differentiation (**C**). Genes with increased expression in *M. bovis* infected cattle relative to controls are shown in red while genes more lowly expressed in this group are shown in green. Shapes represent the downstream protein functions (See appendices for full legend).

Ingenuity Pathway Analysis (IPA) identified the top biological function enriched by differentially expressed genes between groups as lymphocyte activation (*P*-value 6.19E-6, act z-score 2.138) and cellular proliferation (*P*-value 8.07E-11, act. z-score -2.12). On the basis of the differential gene expression, an activation z-score was calculated. It identified that an overall increase in activation (positive value) but reduction of cellular proliferation (negative value) was a likely feature of *M. bovis* infection. The proliferative abilities of CD4^+^ T cells are closely associated with their activation status, which is reflected in the overlapping functional ontology of the genes represented in Fig. 2. Twenty two differentially expressed genes were found to be involved in lymphocyte activation (*P* = 6.76E-5), with 14 of these also found to be involved in T lymphocyte proliferation (including *FASLG* and *GADD45B*) and the expression pattern changes in infected animals relative to controls are shown in Fig. 2A,B, respectively and are listed in Table 1. These genes included the T cell activation marker *CD69* gene and *CD83*, which encode T cell co-receptors. Genes encoding members of the TNF receptor superfamily including *TNFRSF4* and *TNFRSF18* and Ig superfamily members such as *LAG3* and *CD244* were also differentially expressed.

Nineteen genes involved in the process of T cell differentiation were also found to have significantly altered expression patterns (*P*-value 5.77E^−6^, Fig. 2C). Differential expression of genes known to be critically involved in the differentiation of CD4^+^ T cells into their T_H_1, T_H_2, T_H_17 and Treg helper subsets was a consistent finding. Genes encoding master regulators of T helper subsets such *IFNG* (a T_H_1 signature cytokine) and *RORA* (a Th17 specific transcription factor) had increased expression in *M. bovi*s infected cattle. *GATA2*, a member of the GATA family of zinc-finger transcription factors involved in the development and proliferation of hematopoietic cell lineages was significantly decreased in expression in TB infected cattle. These genes, along with others known to be involved in the differentiation of specific T helper cell subsets, were more highly expressed in the infected group of cattle are listed in Table 2.

### Reduced representation bisulphite sequencing (RRBS) of CD4^+^ T cells identifies differential methylation of genomic regions regulating T cell development and differentiation

**Figure 3:**
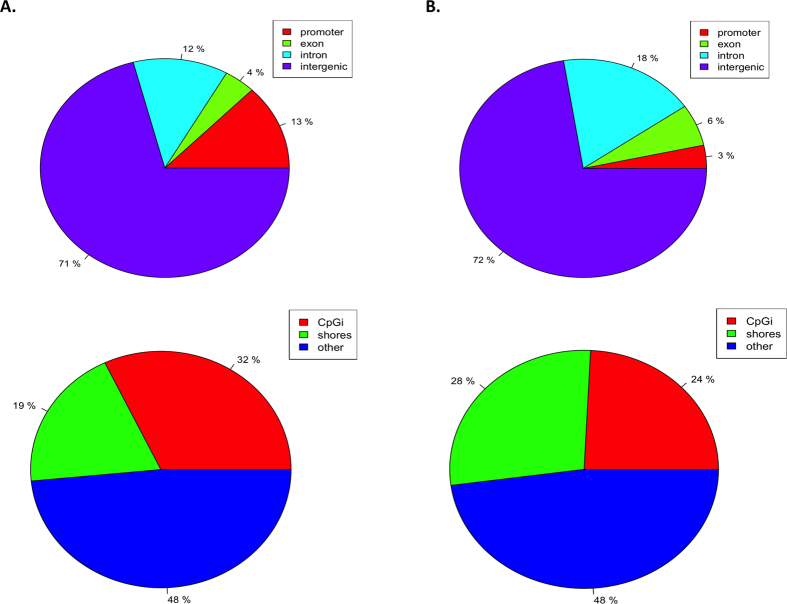
Distribution of differentially methylated CpG sites across genomic features. The proportion of sequenced CpG sites found to be located within promoters, and near or within genes and CpG islands is shown in panel **A**. The distribution of differentially methylated sites found within these regions is shown in panel **B**.

This study represents the first application of the RRBS method (using both Msp1 and Taq1 restriction enzymes) in the bovine. An average of >35 million 50 bp paired-end reads were generated from each bisulphite-converted CD4^+^ T cell DNA sample and the percentage mapping efficiencies ranging from 25.5% to 49.9% - for mapping statistics see Table S3. Of all CpG sites sequenced, the majority (97%) were found to have a sequence depth of between log_10_1 and log_10_2 (Figure S3a). Enrichment of these regions was successful, with 78% of genes in the bovine genome covered by >10 reads. This figure was lower for CpG islands and promoters, with a total of 56% and 47% covered by >10 reads respectively. The majority of genes, promoters and CpG islands sequenced were covered more densely, with many of these features covered by >50 reads (Figure S3b). Of all of the 2675 sequenced CpG sites, 29% were found to be within gene bodies or promoter regions, with the remaining sites (71%) found in intergenic regions (Fig. 3A, upper panel). While 32% of these CpG sites are found within CpG islands, a larger proportion (48%) of sequenced CpG sites are outside of these regions (Fig. 3A, lower panel).

Of all the CpGs found to be differentially methylated, only 3% were located within gene promoters and the majority of CpG methylation data was found within 5 kb of a Transcription Start Site (TSS) [Figure S4]. A further 24% in gene bodies (exons/introns) and again the majority (73%) found within intergenic regions (Fig. 3B, upper panel). 24% of these were located in the context of CpG islands and 28% were found in CpG island shores (2 kb flanks of CpG islands) [Fig. 3B, lower panel]. These findings are consistent with several other studies which have indicated that methylation changes occur more often at CpG island shores, rather than in regions of high CpG density, as was previously thought^21^. The distribution of all differentially methylated CpG sites across the genome is shown in Figure S5. As expected, the differentially methylated sites occur in GC rich regions on each chromosome.

Methylation levels were compared between all biological replicates and pair-wise Pearson correlation scores were calculated for all samples. The enriched methylation analysis identified that DNA methylation patterns are remarkably similar in CD4^+^ T cells, across all samples. Correlation coefficient values ranged from 0.96–0.98 between samples indicating high concordance in methylation pattern between *M. bovis* infected cases and controls at the genome wide level (Figure S6).

Despite the overall similarity in methylation profile, a substantial number of differentially methylated sites and regions were identified. After logistic regression analysis and multiple testing correction, sites/regions that had a q value <0.01 and a percentage change in average methylation of >25% between HC and TB infected groups were identified as statistically significant. Using these criteria, a total of 2675 individual CpG sites were found to be differentially methylated (Table S4). High variation in the magnitude of methylation differences were detected, with a median change of 26% between groups (Table S4). Sliding window analysis to assess methylation levels across 1 kb regions of the genome found a total of 760 windows that were significantly differentially methylated (Table S4), referred to as differentially methylated regions (DMRs). The direction of change was almost evenly divided between DMRs with increased methylation in TB infected cattle (375 DMRs) and those decreased in TB infected cattle (385 DMRs) compared to HC. While gains and losses of DMRs were located across all chromosomes, of note was the presence of 14 DMRs with significantly increased methylation levels on chromosome 6 in TB infected cattle. In contrast to all other chromosomes, no DMRs were reduced in methylation. The magnitude of methylation differences between TB infected and HC groups, across all DMRs reached, a maximum of 77% (Table S4). This analysis revealed differential methylation in regions of the genome proximal to genes known to be crucial for T cell differentiation. This included genes which encode the transcription factors ThPOK and RUNX3; master regulators of CD4^+^/CD8^+^ T cell lineage commitment. The *ZBTB7B* gene, which encodes the CD4-lineage transcription factor ThPOK, was found to be hypermethylated in the *M. bovis* infected animals relative to healthy controls. The CD8-lineage transcription factor *RUNX3* was also identified and found in this case to be hypomethylated. Differentially methylated sites were also found associated with genes important for T helper cell differentiation, including *GATA3* and *RORC* that encode for the master regulators of T_H_2 and T_H_17 subset identity. Upstream of *GATA3* was found to be significantly hypomethylated whereas a region upstream of *RORC* was significantly hypermethylated in *M. bovis* infected cattle.

Genes located within the 760 DMRs (±1 kB) were used for pathway analysis to identify particular pathways and functions associated with the observed DNA methylation changes. Twenty-five genes with differential DNA methylation were associated with the process of T cell development, making this a significantly enriched biological process in this dataset (*P* = 4.9E-03). More specifically, 19 genes were found to be involved in T cell differentiation (*P* = 6.75E-03) and 11 in T helper cell differentiation (*P* = 7.73E-03). Genes involved in these processes are listed, along with their percentage change in average methylation between HC and *M. bovis* infected groups (Table 3). The pathway analysis performed on genes proximal to DMRs shows high concordance with the enriched pathways identified from the gene expression results.

**Figure 4:**
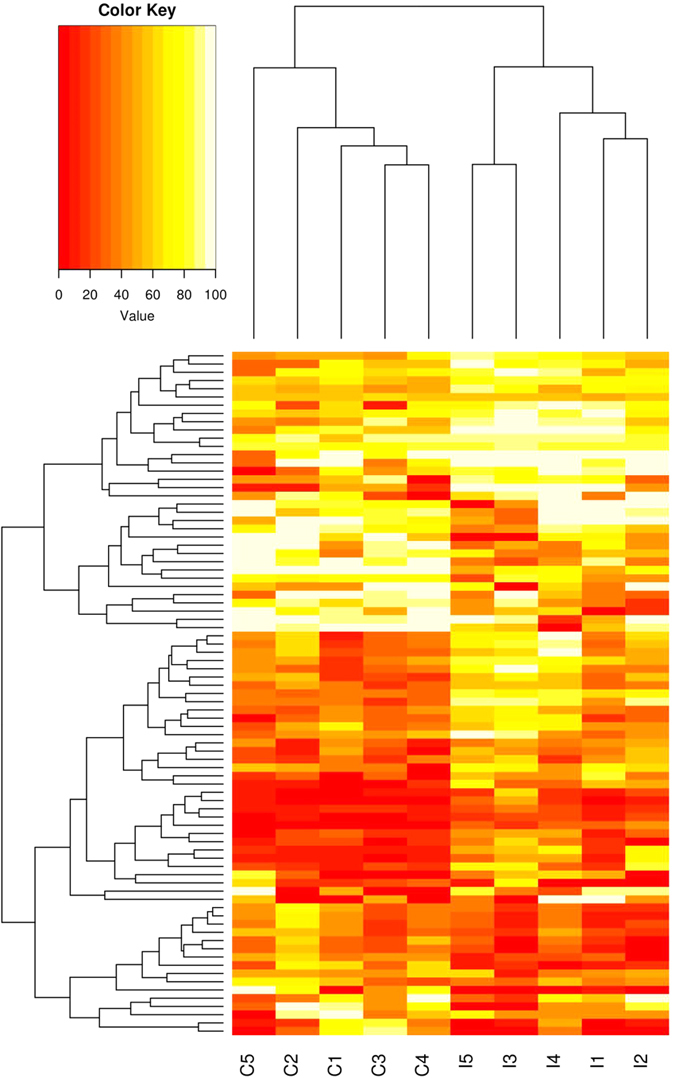
Hierarchical clustering of all control (C) and BTB-Infected (I) samples on the basis of differential methylation profile. A heatmap of 84 CpG sites with methylation levels associated with TB infection status. Each included site is covered by methylated DNA sequence reads in all TB infected and control samples. Each row represents a CpG site, with columns corresponding to individual animals. Higher methylation levels are depicted in yellow and lower levels are red. The upper dendrogram illustrates the results of unsupervised hierarchical clustering of all 10 samples based on methylation patterns at these 84 CpG sites. A clear distinction of controls (labelled C) and infected animals (labelled I) is observed.

From the 2675 differentially methylated CpG sites between groups, a total of 84 sites were represented by DNA reads in all TB-infected and control cattle. Clustering on the basis of these sites shows clear patterns of differentially methylated CpG sites between groups (Fig. 4). These 84 CpG sites occurred within loci encoding 12 annotated genes and 5 microRNAs (Table S4). The analysis of DMRs also encompassed DNA predicted to encode an additional 6 microRNAs. All CpG sites encoding these miRNA are hypomethylated in BTB infected cattle, except sites encoding bta-mir-345 and bta-mir-2387. The full list of predicted differentially methylated microRNA sites is shown in Table 4.

### Negative correlation between expression and methylation levels for TNFRSF4 and IFNG revealed by integrated transcriptomic and methylomic analysis

In order to investigate the likelihood of DNA methylation differences accounting for the observed gene expression differences in *M. bovis* infected cattle, a comparison of differentially expressed and differentially methylated genes was performed. Although DNA methylation changes had been found across the genome and often outside of gene bodies, only differentially methylated sites/regions (q < 0.01, change >25%) found within 50 kb of the transcription start site of a gene were compared. These sites are more likely to have a biological relevance in the context of regulating proximal gene expression. In comparing this list of genes to the list of differentially expressed genes (FDR <0.1), 17 genes were found to be common to both datasets (Table 5). Included in this gene dataset is the *GADD45B* gene which regulates T-cell proliferation and susceptibility to activation-induced cell death. It had increased expression and lower corresponding levels of DNA methylation upstream of the transcription start site in *M. bovis* infected cattle.

**Figure 5:**
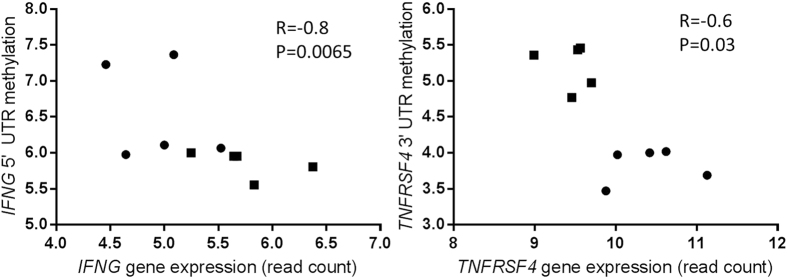
Correlation between gene expression and methylation levels in the (**a**) 5′UTR promoter of the *IFNG* gene and (**b**) 3′UTR of the *TNFRSF4* gene. Log_2_ transformed relative methylation levels were plotted against log_2_ transformed mRNA read counts for 10 biological replicates. Control animals (n = 5) are represented by circles and infected animals (n = 5) are represented by squares. Spearman’s rank correlation test reported a statistically significant negative correlation.

In addition to transcription factors involved in T helper cell differentiation, the expression of the T_H_1 cytokine gene IFNG was significantly upregulated in *M. bovis* infected animals. However, due to the selective nature of RRBS data involving preferential sequencing of CpG rich regions, the IFNG promoter region was not covered by any RRBS reads. Therefore a separate targeted, quantitative qMethyl assay, was used to investigate DNA methylation at the IFNG promoter across four CpG sites. DNA methylation levels at the IFNG 5′ promoter were significantly lower in CD4^+^ T lymphocytes during infection with *M. bovis* (Fig. 5a). IFNG promoter methylation levels were highly similar among all infected samples whereas the variation was higher amongst the control group. As DNA methylation in promoter regions is thought to regulate transcription, a correlation analysis was carried out between gene expression and promoter DNA methylation levels for IFNG. A significant negative correlation was observed with an r value of −0.8, confirming an inverse relationship between IFNG promoter methylation and IFNG expression (*P* < 0.01, Fig. 5a).

Interestingly, the *TNFRSF4* gene, which is an important regulator of T lymphocyte proliferation was also identified as both significantly differentially methylated and differentially expressed in this study. The *TNFRSF4* gene encodes the OX-40 co-receptor, was expressed at significantly lower levels in *M. bovis* infected cattle, and was also significantly hypermethylated at a CpG site in its 3′ untranslated region (UTR). The methylation at this site was on average 41% higher in the lymphocytes of *M. bovis* infected cattle relative to the healthy controls. A significant negative correlation was observed with an r value of −0.6, confirming an inverse relationship between TNFRSF4 3′UTR methylation and gene expression (*P* < 0.05, Fig. 5b).

### TGF an important regulator of expression changes in CD4^+^ T lymphocytes in cattle with BTB

**Figure 6:**
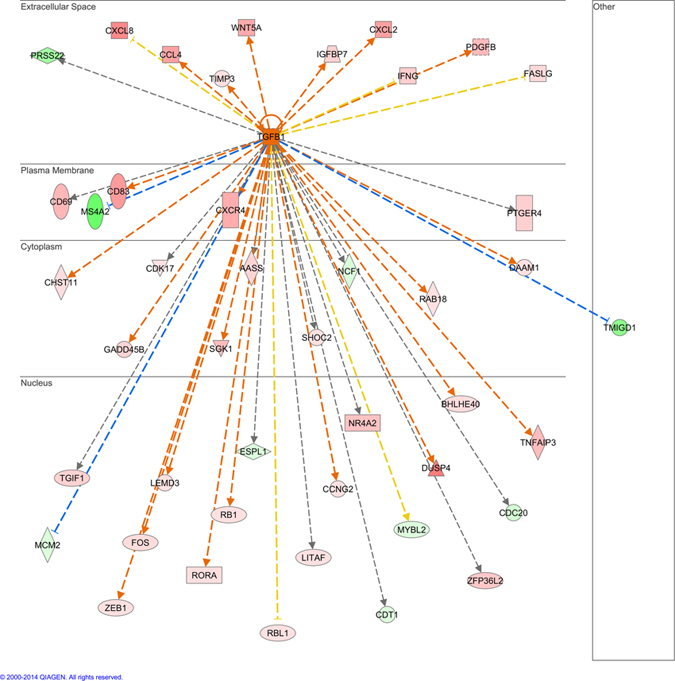
*TGFB1* has been shown to regulate the expression of 46 of the differentially expressed genes in this dataset. Bioinformatic analysis with IPA software identified *TGFB1* as a regulator of genes that were differentially expressed in T lymphocytes from *M. bovis* infected cattle. Gene products are positioned according to their sub cellular localisation in the extracellular space, plasma membrane, cytoplasm and nucleus. Genes up regulated are shown in red, down-regulated genes are shown in green.

Pathway analysis of differentially methylated genes predicted *TGFB1* as a potential regulator of genes involved in regulating CD4^+^ lymphocyte responses during *M. bovis* infection (*P* = 7.95 × 10^−8^). The *TGFB1* gene encodes for the cytokine TGF-β, a protein that controls proliferation and cellular differentiation in immune cells. A total of 46 of the differentially expressed genes have been previously shown to be regulated by *TGFB1* (Fig. 6) and *TGF-β signaling* was found to be a significantly enriched pathway of differentially methylated genes. Significantly hypomethylated regions in the *M. bovis* infected animals were identified near or within the *SMAD2*, *SMAD6* and *SMAD7* genes. SMAD genes encode intracellular proteins that transduce extracellular signals from TGF-β ligands to the cell nucleus. Whilst the TGF-β family of structurally related cytokines induces a multitude of effects in many different cell types, in CD4^+^ T lymphocytes this pathway is important for T cell proliferation, homeostasis and in the differentiation of helper and regulatory T cells.

**Figure 7:**
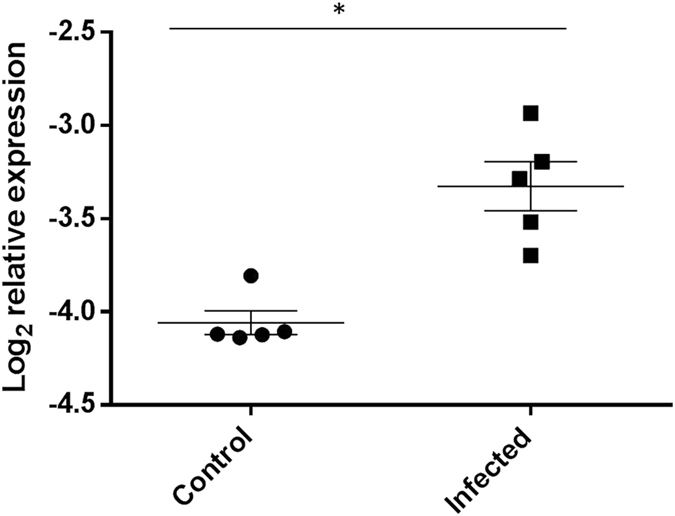
Significantly increased TGFB1 gene expression in cattle infected with *M. bovis.* RNA was extracted from the blood of the *M. bovis* infected (n = 5) and healthy control (n = 5) animals. qRT-PCR analysis was carried out and gene expression data is shown as log_2_ relative expression levels relative to the normalizer gene. A *P* < 0.05 calculated by Mann-Whitney U test was denoted as being statistically significant.

On the basis of the expression profiles of these genes, TGF was predicted by IPA analysis as a core upstream regulator of the observed differential gene expression profiles detected (IPA activation z score = 4.072). However, the corresponding RNA-seq analysis found that the expression of *TGFB1* was unchanged in the CD4^+^ T lymphocytes from the *M. bovis* infected cattle. Subsequent investigation then focused on extra-T lymphocytic sources of this regulatory cytokine. We hypothesized that its expression may have been altered in another circulating leukocyte population in the infected animals, where the cytokines release would affect the proliferative abilities of the circulating CD4^+^ T lymphocytes. qRT-PCR was performed on RNA extracted from peripheral blood leukocytes extracted from whole blood of the same cattle, using primers that specifically targeted the *TGFB1* transcript (Table S5). Results showed significantly higher expression of *TGFB1* was found in the peripheral blood leukocytes from the *M. bovis* infected cattle (*P* < 0.05, Fig. 7).

## Discussion

### Activated but non-proliferative T cells contribute to distinct CD4^+^ T cell signature associated with BTB infection

Despite having the same overall numbers of lymphocytes, CD4^+^ T cells from TB infected cattle exhibit distinct transcriptomic expression profiles, as evident by the clear separation of both groups on the multi-dimensional scaling plot. The collective results of gene expression and pathway enrichment analyses suggest that the processes of T cell activation, proliferation and differentiation are most significantly perturbed in response to BTB infection. Increased expression of genes encoding T cell co-receptors *CD69*-an early inducible cell surface glycoprotein acquired during lymphoid activation and *CD83* that confers immunosuppressive functions to CD4^+^ T cells^22^ was detected in TB infected cattle. The gene encoding the intracellular ubiquitin-editing protein A20, also known as the *TNFAIP3* gene is a key player in the negative feedback regulation of NF-kB signaling in response to multiple stimuli^23^,^24^ was also upregulated. Genes encoding Dual Specificity Phosphatases (*DUSP4* and *DUSP5*) which are known to have a role in TLR signaling^25^ were upregulated, and DUSP4 has previously been shown to suppress T-cell proliferation^26^. *GADD45B* expression, which is associated with induction of T_H_1 cells has also been shown to limit their proliferative abilities and promote apoptosis of activated CD4^+^ T cells^27^ was also increased in TB infected cattle. Some of these genes encode for transcription factors important for cellular differentiation including GATA2 and NFATC3 as well as enzymes such as RAG2, which is critical for lymphocyte development^28^. The *RORA* gene, which encodes for the T_H_17 specific transcription factor RORα was also expressed at significantly higher levels in CD4^+^ T cells from TB infected cattle. In addition, expression of *HOPX*, which has a critical role in inducible regulatory T cell development^29^, was also significantly increased. Enhanced expression of these genes may represent an attempt by the host to limit excessive inflammation associated with tissue damage and to maintain T cell homeostasis via increased apoptosis. However, these findings may also indicate a dysfunctional cellular immune response in the chronic stages of infection. Our data supports findings in humans, where it has been shown that CD4^+^ T cells had profoundly impaired functional capacities in individuals with TB, including severely diminished proliferative abilities^7^.

### Differential methylation of specific regions regulates CD4^+^ T cell fate and function

In recent decades, the T_H_1/T_H_2 paradigm has been expanded to accommodate the discovery of differentiated subsets of CD4^+^ T cells including proinflammatory T_H_17 cells and regulatory T cells (Tregs). T_H_17 cells have been reported to contribute to granuloma formation and the development of a protective adaptive immune response to mycobacterial infection^30^,^31^. Conversely, regulatory T cells and their associated cytokines suppress the protective inflammatory T cell-mediated immune responses during active tuberculosis^32^,^33^, but are thought to contribute beneficial roles by preventing inflammation-mediated damage to host tissues^34^. Whilst these subsets develop from the same CD4^+^ T lymphocyte precursors, their diverse phenotype and function mean that their presence and relative proportions can greatly influence the outcome of infection. As a result, there is increasing interest in the molecular mechanisms underpinning T cell subset differentiation and function across all species^35^. DNA methylation is a mechanism that can lead to robust and epigenetically heritable gene silencing, thereby altering gene expression of genes important for T lymphocyte differentiation. The *GATA3* gene, for example, has been shown to be hypomethylated in T_H_2 cells relative to T_H_1 cells^36^. Similarly, the gene encoding the T_H_17 specific transcription factor Rorτ has been shown to be hypomethylated in T_H_17 cells relative to T_H_1 cells^37^. Therefore, through the regulation of gene expression, epigenetic processes contribute to the development, stability and plasticity of T cell subsets^38^.

In this study, we obtained enrichment of CpG containing DNA across the genome, and detected differentially methylated sites on each chromosome. For some chromosomes, we observed a trend of higher number of differentially methylated sites in telomeric regions corresponding to regions with higher CpG island density, as has been seen with other mammalian genomes^39^. 760 DMRs were detected in CD4^+^ T cells between TB infected and healthy control cattle. Genes encoded in the DMRs were functionally associated with the processes of T cell development and differentiation. VIPR2, a gene encoding a receptor for vasoactive intestinal peptide, with a documented role in T cell polarization^40^ was both differentially methylated and significantly decreased in expression in TB infected cattle. The promoter region of RUNX3 has been shown to display cell type specific methylation patterns across a wide range of tissues^41^; and our data supports a previous finding from the human literature, where RUNX3 was shown to be differentially methylated in CD4^+^ T cells from lupus patients^42^. Pathway analysis of both the differentially expressed gene dataset and the genes encoded in DMRs showed high concordance of functional enrichment of genes related to these ontological functions. Integrated analysis of methylation and transcriptomic data identified a limited number of genes that were both significantly differentially methylated and expressed.

Interestingly, results also showed a significant negative correlation between the level of DNA methylation and gene expression levels for two critical cytokines – TNFRSF4 and IFNy. IFNy is the key cytokine associated with driving a protective anti-mycobacterial immune response and in humans, T_H_1 lymphocyte commitment involves demethylation before IFNG expression^43^. Therefore, our data suggests that progressive methylation of this gene could contribute to the shift toward a non-protective T_H_2 response which dominates during clinical disease in cattle. The TNFRSF4 gene encodes the T cell co-receptor. It has been recently recognised that enhancing OX40 signaling leads to enhanced protection against TB, where delivery of an OX40 agonist in conjunction with the BCG vaccine provided superior immunity against *M. tuberculosis* infection in mice^44^. Our data in cattle also supports findings in mice where *TNFRSF4* was shown to be highly expressed and have low levels of DNA methylation in effector and memory T lymphocytes relative to naïve lymphocytes^45^.

Although not captured for RNA sequencing in the current study, the methylation analysis identified CpG sites and DMRs within regions predicted to encode 11 microRNAs. Several studies in other species have found microRNAs regulate T cell differentiation and function^46^ and can affect TB susceptibility^47^. Bta-mir-22 and mir-345 microRNAs have recently been identified as differentially expressed in bovine macrophages in response to *M. bovis* expression^48^, and their differential expression is suggested to regulate host gene expression to enhance pathogen survival. Mir-345 is a methylation sensitive microRNA involved in cell proliferation^49^. It is known to be downregulated in macrophages in response to *M. tuberculosis*^50^, and is hypermethylated in T cells from TB-infected cattle in this study. Interestingly, multiple members of specific microRNA families, including mir-2887 and mir-2904 were identified close to significantly hypomethylated regions in infected cattle. Mir-22 has been shown to have potential as a sensitive biomarker of TB infection in humans^51^, and is located within a significantly DMR in this study. MicroRNAs are predicted to control the activity of more than 60% of protein-coding genes^52^ including those involved in T cell differentiation and our findings suggest that DNA methylation could play an important role in the regulation of microRNA expression during BTB infection. Given the recent explosion of interest in the potential use of microRNAs as potential diagnostics for infectious diseases, methylation of non-coding regions across the genome could be critical determinants of their reliability as prognostic or diagnostic markers^53^, and therefore further investigation of both cellular and circulating miRNAs during BTB infection is warranted.

### Regulatory role for TGF in BTB infection confirmed in circulating leukocytes

Combined transcriptomic and methylation data analysis also suggested an important key regulatory role for TGF in BTB infection, and we confirmed higher circulating levels of this inflammatory cytokine in infected cattle. These results are consistent with our earlier work which showed significantly elevated TGF-β in PBMCs from BTB-infected cattle in response to mycobacterial antigen stimulation^54^. The TGF-β signaling pathway is known to limit CD4^+^ T lymphocyte proliferation by reducing the effects of CD28 co-stimulation^55^ and TGF-β has previously been shown to regulate the lymphocyte response to mycobacterial infection in mice^56^. TGF beta has also been shown to suppress a protective immune response against *M. bovis* by promoting elimination of activated T cells^57^. Furthermore, previous studies in humans have identified higher levels of TGF-β production by circulating monocytes in tuberculosis patients and that this increase is a major contributor to depressed T cell functions in peripheral blood^58^,^59^.

## Conclusion

In this study, we have identified a distinctive CD4^+^ T cell transcriptomic signature which clearly segregates BTB infected from HC cattle, indicating clear functional divergence in this key cell subset in response to infection. These results support our earlier work which indicated that BTB induces a distinctive gene expression signature in both peripheral blood cells and in macrophages^60^. However, in line with emerging related work on human TB^61^, increased expression of the majority of genes in CD4^+^ T cells from TB infected cattle in this study suggests that the widespread immune gene suppression detected in our earlier work is more a feature of the innate immune responses to mycobacterial infection. The moderate, widespread and ontologically-related changes in T cell methylation identified in this study likely contributes to the transcriptomic profile of this CD4^+^ T cell population, both directly through regulation of gene expression levels but also post-transcriptionally via modulation of microRNA expression.

Despite the inherent variation in methylation levels expected under natural infection conditions, combined genome-wide methylation and transcriptomic analyses of bovine lymphoid cells reveals a novel role for DNA methylation in the impaired lymphocyte proliferation during *M. bovis* infection. It is of interest that a negative correlation was detected between gene expression and methylation levels for important cytokines, including IFNγ. As has been shown in studies of TB in humans, it may be the case that the degree of methylation at the IFNG promoter will impact on the immune response profile of individual cattle^62^ and hypermethylation may ultimately contribute to T cell exhaustion or anergy. In addition, the anti-proliferative cytokine TGF-β was identified as a major regulator of lymphocyte function during infection.

These data are relevant to human TB infection and to the study of other related mycobacterial infections in cattle, including Johne’s disease, caused by the related *Mycobacterium avium* subsp. *paratuberculosis*^63^,^64^. We also detected changes in the WC1^+^ γδ T cell population in TB infected cattle link innate and adaptive arms of the immune response and are informative candidates for future research. Integrated approaches will enable the comprehensive understanding of the molecular architecture regulating host responses to mycobacterial infection and transcriptomic signatures based on the emergent T cell response during BTB may aid the development of more sensitive diagnostics. Finally, gene-specific methylation changes at key inflammatory gene loci may be critical to the emergence of a non-protective CD4^+^ T cell response during mycobacterial infection in cattle, and these too may have important future prognostic potential to improve detection and eradication of TB in cattle.

## Materials and Methods

### Experimental animals

Ten age-matched male Holstein-Friesian animals were used for this study. Infected cattle (n = 5) were selected from a herd of *M. bovis* infected animals maintained at the Department of Agriculture research farm in Longtown, Co. Kildare, Ireland. These animals had tested positive for bovine tuberculosis by single intradermal comparative tuberculin test (SICTT), in response to purified protein derivative (PPD)-bovine. These animals also tested positive for tuberculosis using the whole blood IFNγ based BoviGAM assay [Prionics AG, Switzerland). Non-infected, age- and sex-matched control animals (n = 5) were selected from a herd with a TB free history for greater than 8 years. Control animals were shown to be negative for both the SICTT and Bovigam assay. All experimental protocols sampling was carried out in accordance with the relevant guidelines and under ethical approval and license from the Irish Department of Health and Children.

### Blood sampling, processing and CD4^+^ T cell sorting

One 9 ml vacutainer containing EDTA anticoagulant was collected for haematological analysis, using the Advia 2120 Hematology Analyzer (ADVIA 2120, Bayer Healthcare, Siemens, UK). Four additional 9 ml vacutainers containing Heparin anticoagulant were also collected for DNA methylation and gene expression analysis. A Tempus tube containing 3 ml of blood was also collected for RNA extraction from whole blood and stored at −20 °C. Peripheral blood mononuclear cells were isolated from whole blood using the Leucosep system (Greiner Bio-One Ltd. Brunel Way, Stroudwater Business Park, Stonehouse, Great Britain) and Histopaque-1088 (Sigma-Aldrich Ireland Limited, Vale Road, Arklow, Co Wicklow, Ireland). The cells were resuspended in 1 ml PBS containing 2% foetal calf serum (FCS) and labelled with a mouse anti bovine CD4 antibody conjugated to FITC (Clone CC8, AbD Serotec, Endeavour House, Langford Business Park, Kidlington, Oxford, UK). After incubation, cells were gently washed and passed through a sterile filter to remove any clumps. CD4^+^ cells were isolated by fluorescent activated cell sorting with purity greater than 99%. Propidium iodide (Invitrogen/Life Technologies Corp., Paisley, UK) was also used for the exclusion of dead cells and sorted cells were identified as having viability greater than 95%. CD4^+^ T cells were lysed in Trizol (Invitrogen/Life Technologies Corp., Paisley, UK) immediately after sorting and the cell lysates were stored at −80 °C.

### RNA extraction and TruSeq RNA library preparation

RNA was extracted from Trizol using the RNeasy plus RNA extraction kit (Qiagen Ltd., Crawley, UK). The aqueous layer of the Trizol/Chloroform sample was added to 70% ethanol at a ratio of 1:2 and mixed before being added directly to the RNeasy column. RNA was further purified using the RNeasy plus kit as per the manufacturer’s instructions. RNA quantity and quality were assessed using both a nanodrop spectrophotometer and the Agilent 2100 bioanalyzer. All samples were found to have RIN values greater than 8 and were found to be free from contaminating protein and organic compounds. TruSeq (Illumina, Chesterford Research Park, Essex, CB10 1XL, UK.) RNA libraries were prepared from a starting quantity of 250 ng high quality RNA. All libraries were sequenced over 2 lanes of the Illumina, HiSeq 2000, generating 75 bp paired end reads.

### Bioinformatic processing and RNA-seq data analysis

Sequence quality and composition were checked using FastQC (v0.10.0) software and adapter and quality trimming was carried out using Cutadapt (v1.2.1) software. Sequence reads with a Phred score greater than 20 were then mapped to the bovine genome (BosTau7/Btau_4.6.1) using the software tool TopHat (v2.0.8), which utilises Bowtie2 (v2.0.5). After mapping, raw counts per gene were calculated using HTSeq (htseq-count, v0.5.3p3). Counts of uniquely mapped reads were obtained for all bovine genes and transcripts. Calculation of differential expression was carried out using the R statistical package edgeR (v3.6.2). After filtering of lowly expressed reads, TMM normalization was applied to the data to account for compositional difference between the libraries using both common and tag-wise dispersion estimations. The detection of significantly differentially expressed genes (FDR <0.05) was carried out using the exact test in EdgeR. P-values were subsequently adjusted for multiple testing using the Benjamini-Hochberg correction. Pathway analysis was carried out using Ingenuity Pathway Analysis (IPA) and genes differentially expressed with an FDR <0.1 were included. IPA allowed for the identification of molecular and cellular functions known to be associated with the differentially expressed genes in this study.

### DNA extraction and RRBS library preparation

Chloroform was added (1:2 ratio) to the Trizol lysates before centrifuging at 4 °C for 15 min at 13000 x g. DNA was isolated from the phase separated organic layer. DNA was then purified using the Clean and Concentrator kit (Zymo Research, Irvine, CA, USA) and resuspended in a volume of 20 μl. EpiQuest libraries, which were designed to be non-directional, were prepared from 500 ng ultrapure genomic DNA. The DNA was digested with 60 units of *Taq*I and 30 units of *Msp*I (NEB) sequentially. Size-selected *Taq*I-*Msp*I fragments (40–120 bp and 120–350 bp) were filled-in and 3′-terminal-A extended. Ligation to pre-annealed barcoded adaptors containing 5′-methyl-cytosine was performed using the Illumina DNA preparation kit and protocol. Purified, adaptor-ligated fragments were bisulphite treated using the EZ DNA Methylation-Direct(tm) Kit (Zymo Research). Preparative-scale PCR was performed and DNA Clean and Concentrator-purified PCR products were subjected to a final size selection on a 4% NuSieve 3:1 agarose gel. SYBR-green-stained gel slices containing adaptor-ligated fragments of 130–210 or 210–460 bp in size were excised. Barcoded libraries were sequenced across 2 lanes of an Illumina HiSeq 2000, generating 50 bp paired end reads.

### Bioinformatic processing and DNA methylation analysis

Firstly, the quality and composition of the sequence data was checked using FastQC (v0.10.0). The data was then quality and adapter trimmed using Trim Galore (v0.2.8). Reads containing bases with a Phred score of less than 20 were quality trimmed. In addition, the presence of any Illumina adaptor sequences at the end of reads were identified and removed. Quality and adapter trimmed reads were then mapped to the bovine reference genome (BosTau7/Btau_4.6.1) using the software tool Bismark (v0.9.0). Reads that mapped uniquely were maintained whilst reads that mapped to multiple locations were discarded. Alignment parameters were set to allow one mismatch in the seed region, which was defined as the first 20 bp of every read. After mapping, an addition step was taken to ensure that filled in Cs as a result of Taq1 digestion were not used for methylation calling using an in-house perl script. Differential methylation analysis was carried out using the R package methylKit (v0.9.2)^65^. Samples were filtered so that CpG sites that had <5x coverage were excluded from the analysis. Differential methylation analysis between control and infected groups was carried out at single nucleotide resolution as well as across promoter regions (defined as 2 kb upstream of a transcription start site) and 1 kb sliding windows of the genome. CpG sites were required to be covered by at least 5x reads in at least 3 samples per group to be included for statistical analysis. Logistic regression was implemented in methylKit to calculate P values for samples from control versus *M. bovis* infected animals. Multiple testing corrections were carried out using the sliding linear model (SLIM)^66^, producing corresponding q values. In order for a site or region to be classified as differentially methylated, it was required to have a q > 0.01 in addition to a percentage methylation change >25% between control and BTB groups. Differentially methylated sites were annotated using gene and CpG island annotation, downloaded from the UCSC genome browser^67^. GC content across each chromosome was calculated in sliding windows of 10 Kb, moving 1 Kb, using the Isochore tool within Emboss^68^. Pathway analysis was carried out using Ingenuity Pathway Analysis (IPA; Ingenuity Systems, Redwood City, CA, USA; www.ingenuity.com; v18030641) using genes located closest to differentially methylated sliding windows.

### Primer design and qMethyl PCR amplification of the IFNG promoter

Quantitative methylation analysis at the IFNG gene was performed using a combination of restriction digestion and real time PCR, utilised as part of the qMethyl (Zymo Research Corp, Irvine, California, USA) protocol. Primers were designed to span a 303 bp region of IFNG, which contained 4 methylation sensitive restriction enzyme sites (as shown in Table S5). Briefly, DNA from each sample was split so that two different reactions could be carried out- the test and reference reactions (20 ng/reaction). Only the test reaction DNA is digested with methylation sensitive restriction enzymes. Each DNA sample was added to a well of a 96 well plate containing 10 μl 2x test/reference reaction mix, 1 μl each of forward and reverse primers (10 μM) and 3 μl H2O. After digestion, the IFNG promoter region is amplified by RT-qPCR. The Ct value differences between the test and reaction samples will depend on the methylation status of the promoter. PCR amplifications were carried out using the Applied Biosystems 7500 Fast v2.0.1 real time PCR instrument. Data was analysed using an online analysis tool called qMethyl calculator (Zymo Research Corp, Irvine, California, USA) and results were displayed as relative methylation changes. Mann Whitney U tests were used to statistically analyse methylation levels between groups.

### RNA extraction from whole blood

RNA was extracted from Tempus blood RNA tubes (Applied Biosystems, Warrington, UK) for all animals included for RNA-seq and RRBS of CD4^+^ lymphocytes. RNA was purified using the Tempus Spin RNA Isolation Kit (Applied Biosystems, Warrington, UK) following manufacturer’s guidelines.

### cDNA synthesis, primer design and qRT-PCR

All RNA samples were diluted to a concentration of 50 ng/μl in a final volume of 10 μl. The High-Capacity cDNA Reverse Transcriptase kit (Applied Biosystems, Warrington, UK) was used to convert RNA to cDNA as per manufacturer’s instructions. Primers were designed using the UCSC bovine genome browser, Primer 3 (Version 0.4.0) and checked for specificity using Primer Blast (NCBI). All primers used were also designed to be intron spanning and were commercially synthesized (Sigma–Aldrich Ireland Ltd., Wicklow, Ireland). RT-qPCR was performed using the SYBR green-based fluorescent method and using the Applied Biosystems Fast 7500 v2.0.1 instrument. qRT-PCR data were converted to 2^−deltaCt^ values, expressed relative to the lowest value and log_2_ transformed for graphical representation and statistical analysis. This was carried out using GenEx 5.2.1.3 (Multi D Analyses, Gothenburg, Sweden) and statistical analysis was carried out using Graphpad Prism 5 (Mann Whitney U test).

## Additional Information

**How to cite this article**: Doherty, R. *et al*. The CD4^+^ T cell methylome contributes to a distinct CD4^+^ T cell transcriptional signature in *Mycobacterium bovis*-infected cattle. *Sci. Rep.* **6**, 31014; doi: 10.1038/srep31014 (2016).

## Supplementary Material

Supplementary Figures

Supplementary Tables

Supplementary Table S2

Supplementary Table S4

## Figures and Tables

**Table 1:** Pathway analysis identified a significant enrichment of genes involved in the process of T lymphocyte proliferation.

Gene ID	Direction of change in BTB infected cattle	P value	FDR
*MIF*	Decreased	0.001516006	0.077852641
*DTX1*	Decreased	1.40E-06	0.000895069
*LAG3*	Decreased	6.43E-05	0.013256744
*TNFRSF4*	Decreased	9.12E-06	0.003604011
*TNFRSF18*	Decreased	0.000775883	0.053792241
*PSMB10*	Decreased	0.001647765	0.081387012
*CD226*	Increased	0.000648583	0.04917853
*PAWR*	Increased	0.001159251	0.0649226
*CD244*	Increased	0.002033674	0.090686545
*BHLHE40*	Increased	9.86E-05	0.017051434
*FOS*	Increased	0.002381354	0.099925563
*FASLG*	Increased	0.000275069	0.030829375
*ADRB2*	Increased	0.000657992	0.04917853
*GADD45B*	Increased	0.000581126	0.045671392
*PTGER4*	Increased	0.000622163	0.04783926
*IFNG*	Increased	0.000711758	0.051656956
*SOX13*	Increased	2.83E-05	0.008062212
*TNFAIP3*	Increased	5.36E-05	0.012922284
*CD69*	Increased	1.02E-05	0.00393129
*CXCR4*	Increased	0.000199197	0.025996169
*RAG2*	Increased	0.001452816	0.075654384
*CCL4*	Increased	4.79E-06	0.002271753
*CD83*	Increased	6.40E-05	0.013256744

**Table 2:** List of genes known to be crucial for CD4^+^ T cell differentiation into effector T cell subsets that were differentially expressed between BTB infected and healthy control cattle.

Gene ID	FDR	Direction of change in BTB infected cattle	Associated Th subset
*IFNG*	0.051656956	Increased	Th1
*IL18RAP*	0.039040465	Increased	Th1
*FASLG*	0.030829375	Increased	Th1
*RORA*	0.066664349	Increased	Th17
*TGIFI*	0.004451754	Increased	Treg
*HOPX*	0.05258242	Increased	Treg
*TP53INP1*	0.01867168	Increased	Treg

**Table 3:** List of genes known to be crucial for CD4^+^ T cell differentiation into effector T cell subsets during TB infection closest genes to significantly differentially methylated sites/regions.

Gene ID	Analysis Type	Percentage change in BTB infected cattle	Associated Th subset
NFATC1	1 kb window	−32.5	Th2
ASB	1 kb window	−28.4	Th2
GATA3	1 kb window	−41.9	Th2
IRF4	Individual CpG site	31.9	Th2/Treg
RORC	1 kb window	29.7	Th17
HOXA10	Promoter region	−39.3	Treg
LRRC32	1 kb window	40.4	Treg
ZBTB7B/ThPOK	1 kb window	34.5	Promotes CD4^+^ differentiation
RUNX3	1 kb window	−40.9	Inhibits CD4^+^ differentiation, promotes Tregs

**Table 4:** List of microRNAs encoded within differentially methylated regions (±1 kB).

MicroRNA	Percentage change in methylation in BTB cattle	Method
Bta-mir-22	−34.9	DMR
Bta-mir-345	27.2	DMR
Bta-mir-2284z-2	−33.7	DMR
Bta-mir-2387	32.3	DMR
Bta-mir-2461	−37.3	DMR
Bta-mir-2887-1	−27.9*	CpG site
Bta-mir-2887-2	−27.9*	CpG site
Bta-mir-2904-1	−27.9*	CpG site
Bta-mir-2904-2	−27.9*	CpG site
Bta-mir-2904-3	−27.9*	CpG site
Bta-mir-3600	−34.9	DMR

**Table 5:** List of differentially expressed genes located proximally to a differentially methylated site/region.

DE genes with neighbouring DM site	DE genes with neighbouring DM region
VIPR2	VIPR2
TNFRSF4	PYCR1
SLC29A4	DAK
ATP4B	STARD10
ISLR	CENPM
EEFSEC	EIF5
GATA2	GADD45B
	SDR42E1
	CA5A
	PHYHD1
	THBS2

Differentially expressed genes (FDR <0.1) and differentially methylated (DM) genes (q < 0.01, change >25%, within 50 kb of TSS) were compared to identify genes common to both datasets. Differentially methylated sites refer to individual CpG sites where as differentially methylated regions refer to 1 kb regions of the genome.
