# Supplementary material for: The CD4+ T cell methylome contributes to a distinct CD4+ T cell transcriptional signature in Mycobacterium bovis-infected cattle
Source: Sci Rep. 2016 Aug 10;6:31014. doi: 10.1038/srep31014 (PMC4978967; doi:10.1038/srep31014)
Supplement: Supplementary Figures [file srep31014-s1.pdf]

The CD4$^+$ T cell methylome contributes to a distinct CD4$^+$ T cell transcriptional signature in *Mycobacterium bovis*-infected cattle

Rachael Doherty[1, 2], Ronan Whiston[1, 2], Paul Cormican[1], Emma K. Finlay[1], Christine Couldrey[3], Colm Brady[4], Cliona O'Farrelly[2] and Kieran G. Meade[1].

Supplementary Figures

Figure s1 Hematological analysis comparing the absolute numbers of different leukocyte populations between healthy and BTB infected cattle. The numbers of white blood cells (A), lymphocytes (B), neutrophils (C) and monocytes (D) per microlitre of blood are shown. Differences in the number of cells between control and infected cattle were analysed using a Mann Whitney U test. $P<0.05$ were denoted as being statistically significant.

Figure s2 Measurement of the proportions of T lymphocyte populations in PBMCs. Histograms on left hand side of figure s2 show fluorescence intensity from PBS (unstained control) (A), CD4 (B), CD8 (C) and WC1 (D) positive cells in red and the isotype control in blue. Corresponding dot plots show fluorescence intensity for these markers on the x axis versus propidium iodide on the y axis. The results on the right hand side of Figure s2 show flow cytometric analysis comparing the frequencies of the different T lymphocyte populations between healthy and TB infected cattle. The proportion of PBMCs expressing CD4$^+$ (A), CD8$^+$ (B) and WC1$^+$ (C) are shown. Proportional differences between control and infected cattle were analysed using a Mann Whitney U test. $P<0.05$ was denoted as being statistically significant.

Figure s3(a) Depth of coverage of CpG sites obtained using RRBS to analyse the genome-wide methylation patterns in bovine T lymphocytes. The coverage across the genome identified that the majority of CpG sites covered by sequence reads across the genome had a sequencing depth between 10 and 32.
Figure s3(b) Depth of coverage shown across main genomic features plotted versus the number of genes (A), promoters (B) and CpG islands (C).

Figure s4 Distribution of differentially methylated CpG sites relative to transcription start sites (TSS). The x axis shows the distance from differentially methylated sites to the nearest TSS. The frequency of differentially methylated sites is plotted on the y axis.

Figure s5 Distribution of differentially methylated CpG sites across the genome. Sites which are hypermethylated in BTB-infected cattle are coloured red with hypomethylated sites in green. The average GC content of each 1 Kb genomic window is represented in the lower panel for each chromosome, coloured according to average GC content in that window - GC <40%, white; 40%< GC< 60%, grey and GC > 60%, black.

Figure s6 Scatter plots of % methylation values for each pair of samples. Numbers in upper right corner denote pair-wise Pearson's correlation scores. The histograms on the diagonal are % methylation histograms for each sample showing the typical bimodal distribution observed for DNA methylation. Sample names are denoted on the diagonal.

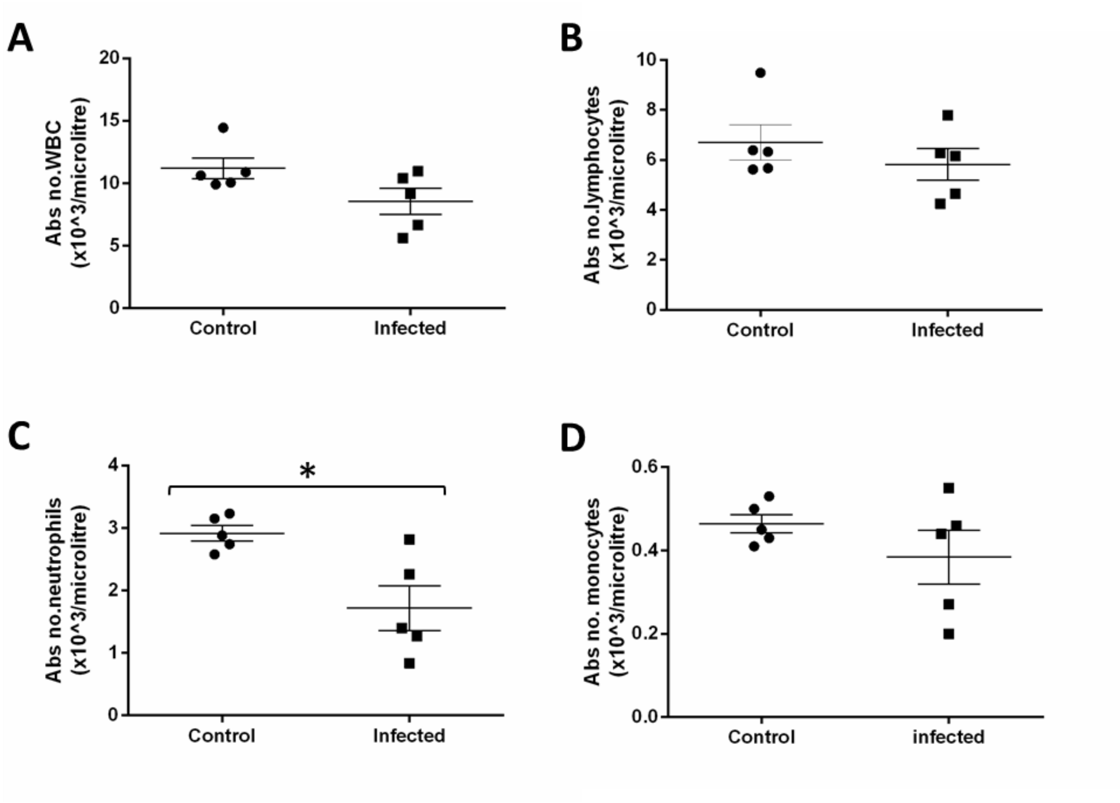

Figure s1 Hematological analysis comparing the absolute numbers of different leukocyte populations between healthy and BTB infected cattle. The numbers of white blood cells (A), lymphocytes (B), neutrophils (C) and monocytes (D) per microlitre of blood are shown. Differences in the number of cells between control and infected cattle were analysed using a Mann Whitney U test. P values < 0.05 were denoted as being statistically significant.

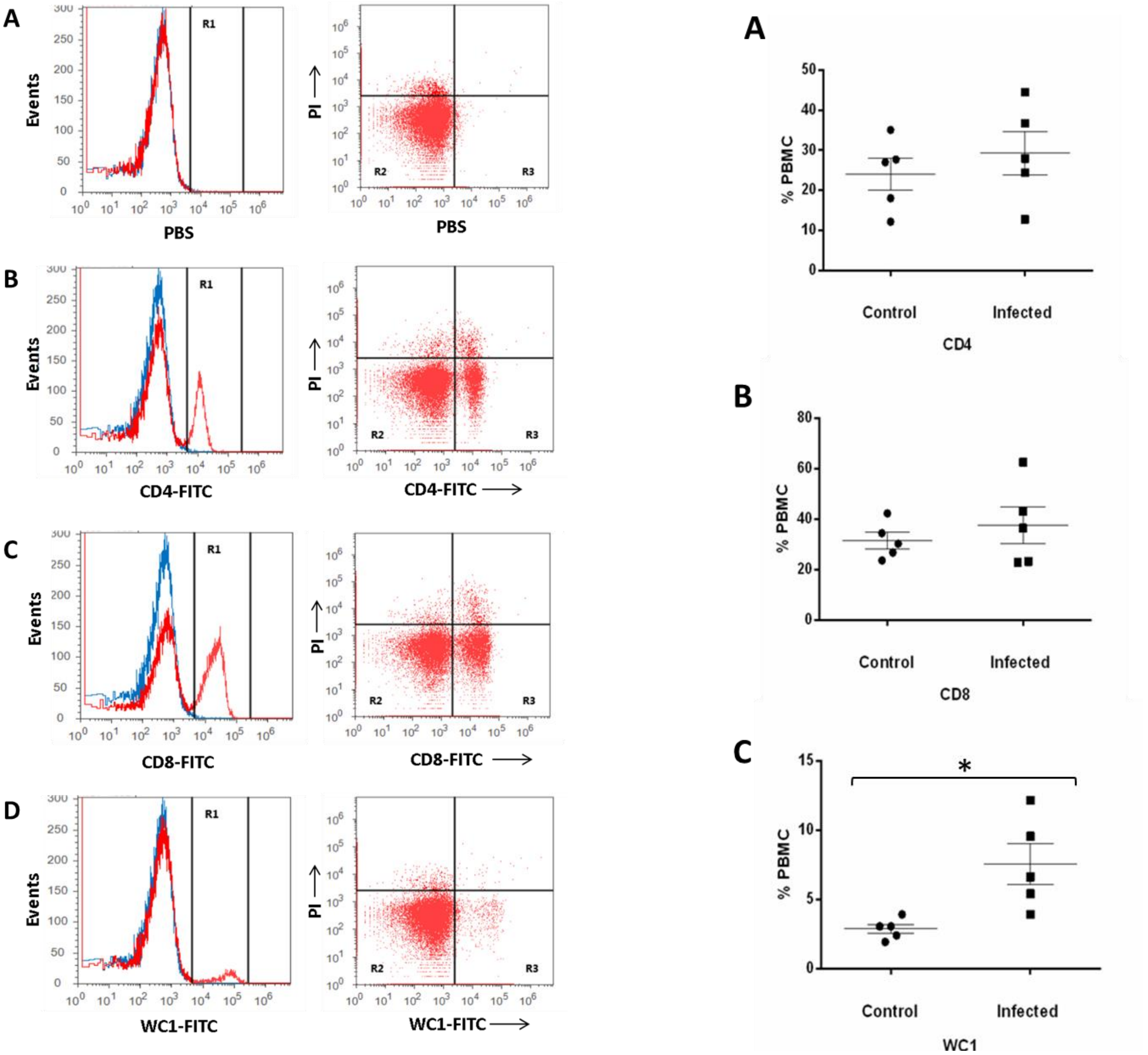

Figure s2 Measurement of the proportions of T lymphocyte populations in PBMCs. Histograms on left hand side of figure s2 show fluorescence intensity from PBS (unstained control) (A), CD4 (B), CD8 (C) and WC1 (D) positive cells in red and the isotype control in blue. Corresponding dot plots show fluorescence intensity for these markers on the x axis versus propidium iodide on the y axis. A total of 10,000 events were collected for each sample and the percentage of cells in R3 of the quadrant were compared between control and infected animals. The results on the right hand side of figure s2 show flow cytometric analysis comparing the frequencies of the different T lymphocyte populations between healthy and TB infected animals. The proportion of PBMCs expressing CD4 (A), CD8 (B) and WC1 (C) was assessed by flow cytometry. Proportional differences between control and infected cattle were analysed using a Mann Whitney U test. P values < 0.05 were denoted as being statistically significant.

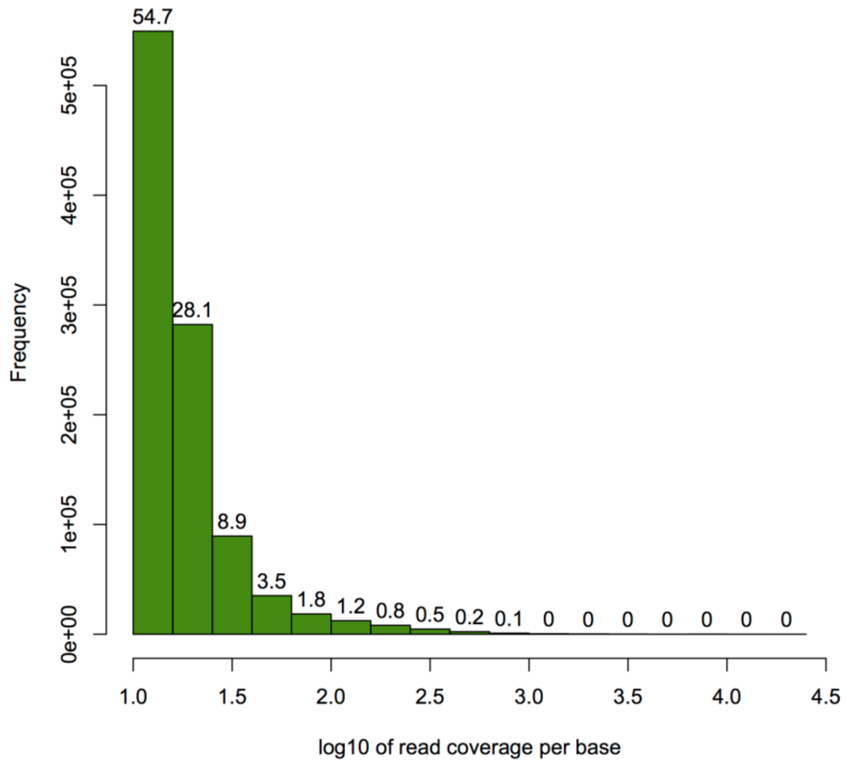

Figure s3(a) Depth of coverage of CpG sites obtained using RRBS to analyse the genome-wide methylation patterns in bovine T lymphocytes. The coverage across the genome identified that the majority of CpG sites covered by sequence reads across the genome had a sequencing depth between 10 and 32.

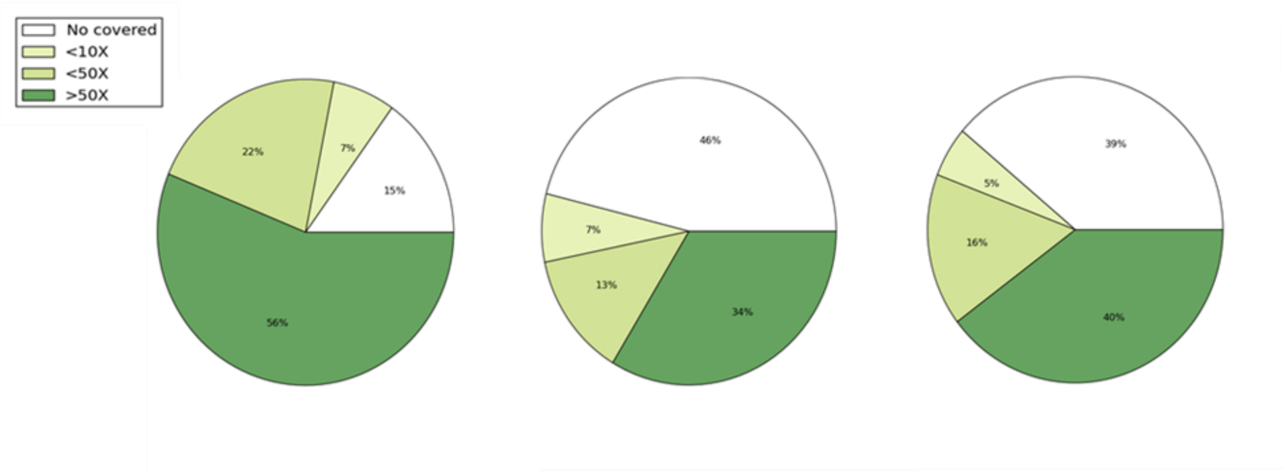

Figure s3(b) Depth of coverage of CpG sites obtained using RRBS to analyse the genome-wide methylation patterns in T lymphocytes from healthy and TB infected cattle

The coverage obtained for CpG sites found within genes, promoters and CpG islands, which are preferentially sequenced using the RRBS protocol are shown.

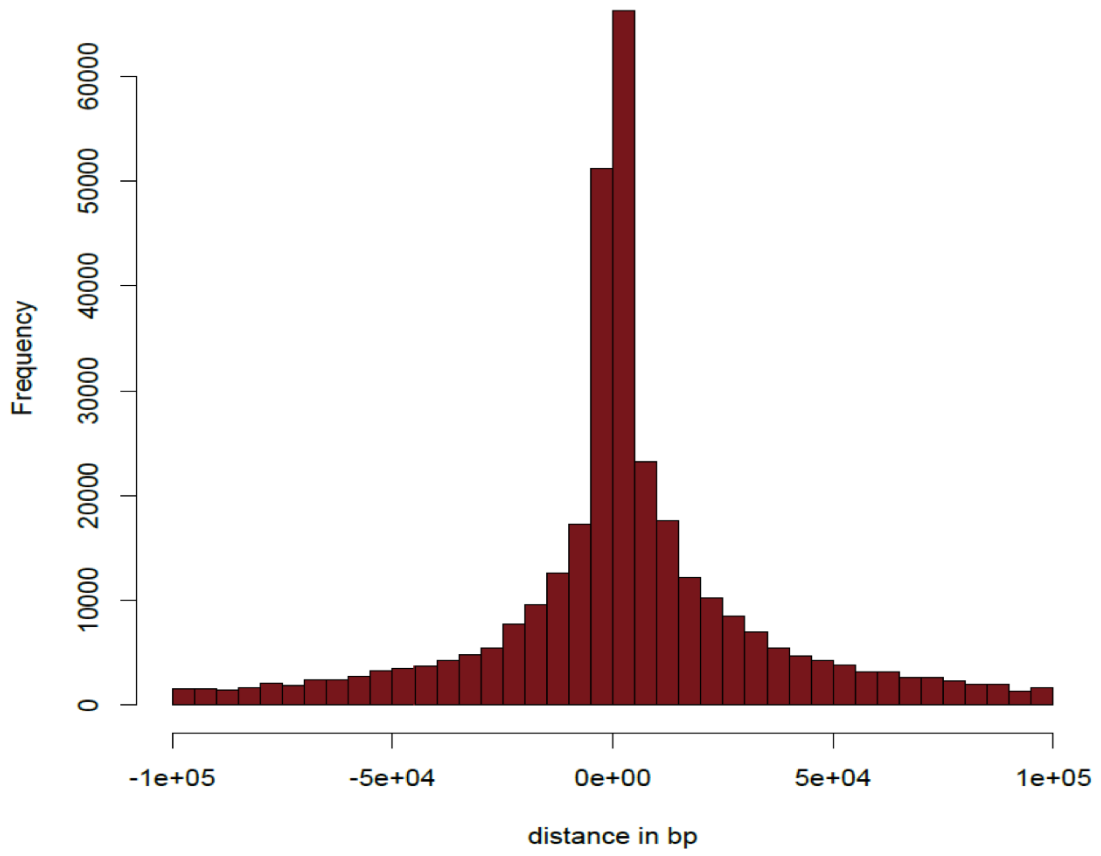

Figure s4 Distribution of differentially methylated CpG sites relative to transcription start sites (TSS). The x axis shows the distance from differentially methylated sites to the nearest TSS. The frequency of differentially methylated sites is plotted on the y axis.

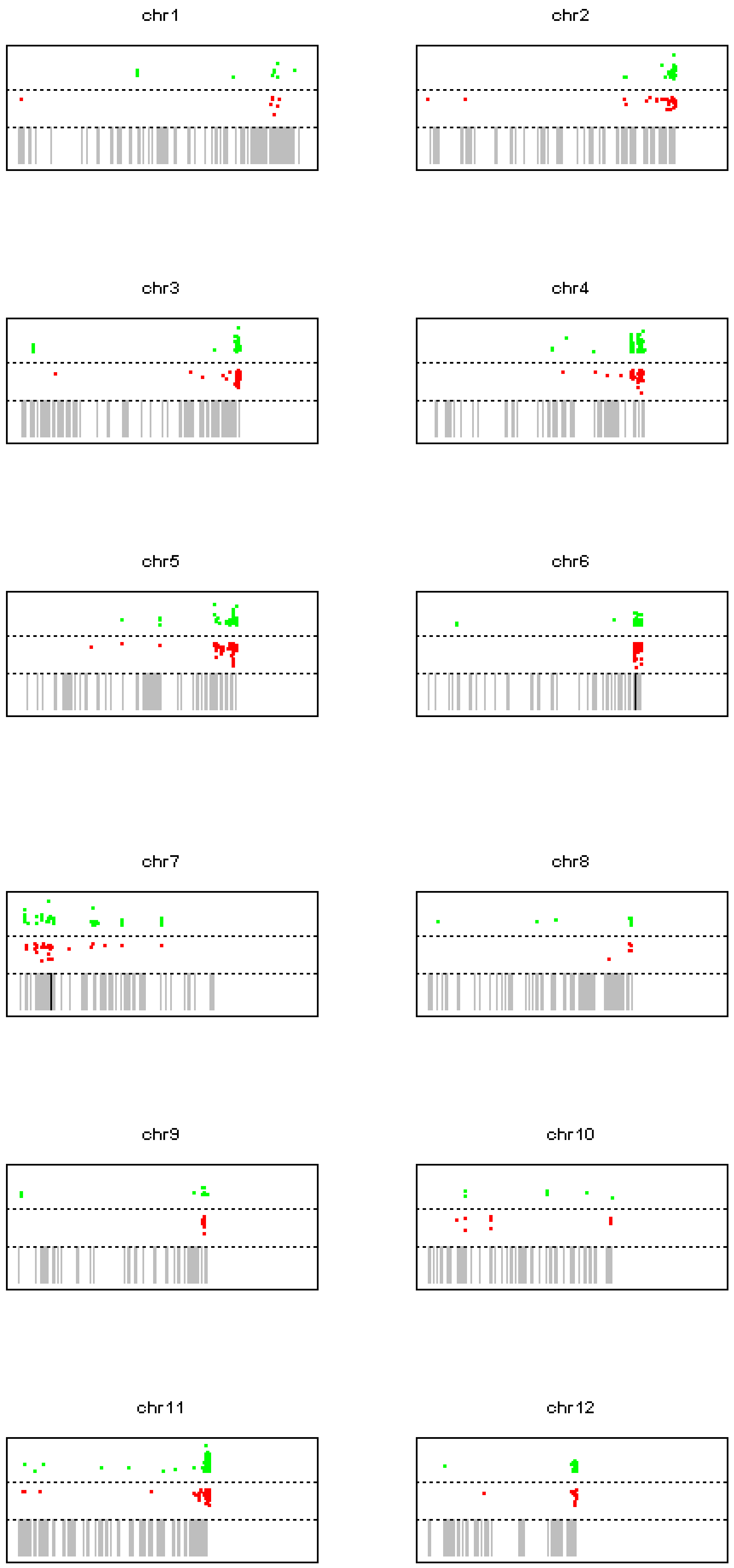
chr1
chr2
chr3
chr4
chr5
chr6
chr7
chr8
chr9
chr10
chr11
chr12

chr13

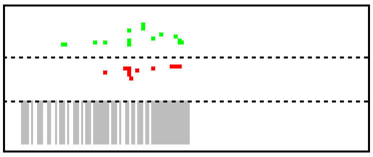

chr14

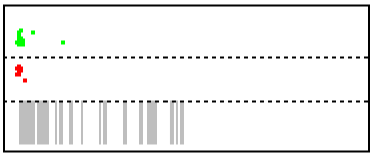

chr15

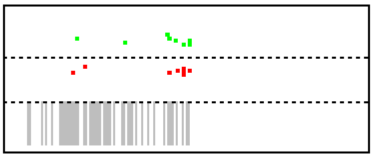

chr16

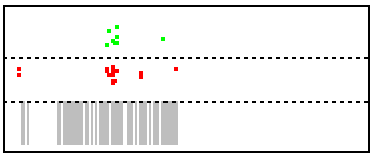

chr17

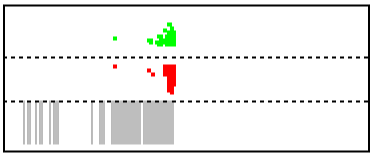

chr18

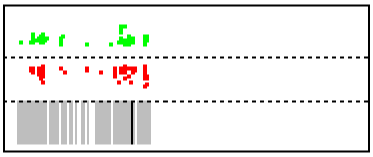

chr19

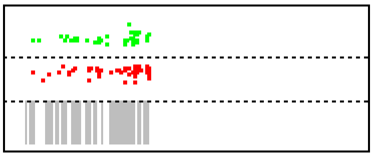

chr20

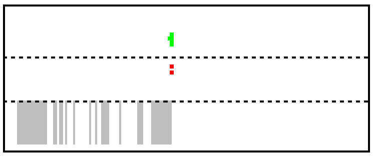

chr21

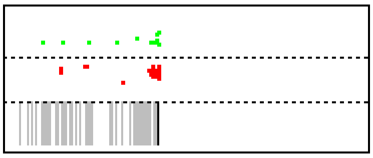

chr22

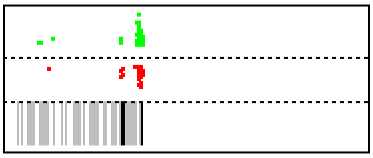

chr23

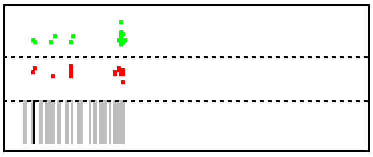

chr24

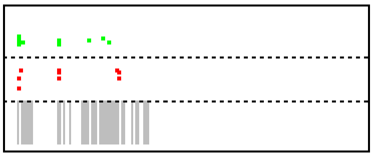

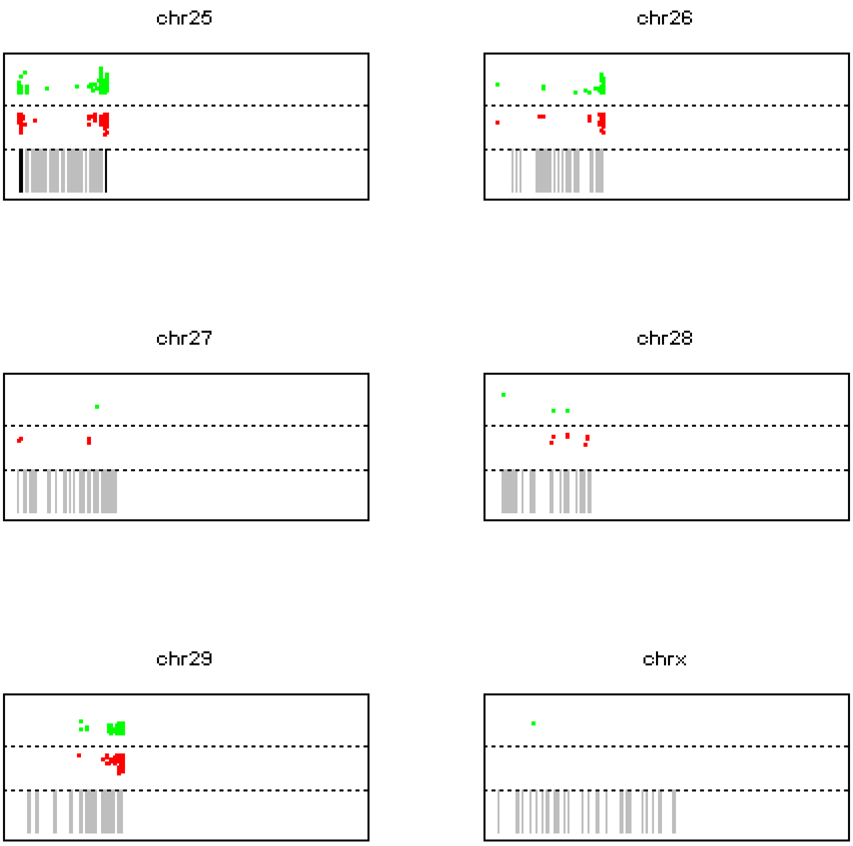

Figure s5 Distribution of differentially methylated CpG sites across the genome. Sites which are hypermethylated in BTB-infected cattle are coloured red with hypomethylated sites in green. The average GC content of each 1 Kb genomic window is represented in the lower panel for each chromosome, coloured according to average GC content in that window - GC <40%, white; 40%< GC< 60%, grey and GC > 60%, black.

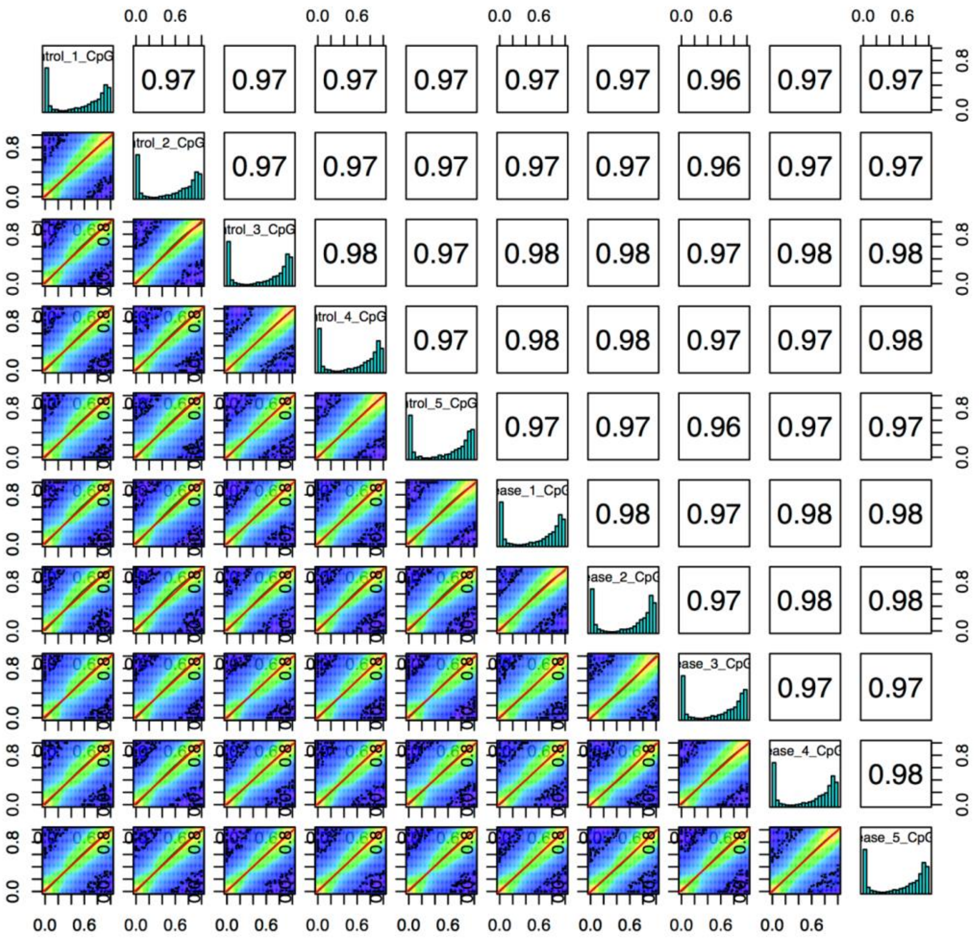

Figure s6 Scatter plots of % methylation values for each pair of samples. Numbers in upper right corner denote pair-wise Pearson's correlation scores. The histograms on the diagonal are % methylation histograms for each sample showing the typical bimodal distribution observed for DNA methylation. Sample names are denoted on the diagonal.
