# Supplementary material for: The CD4+ T cell methylome contributes to a distinct CD4+ T cell transcriptional signature in Mycobacterium bovis-infected cattle
Source: Sci Rep. 2016 Aug 10;6:31014. doi: 10.1038/srep31014 (PMC4978967; doi:10.1038/srep31014)
Supplement: Supplementary Tables [file srep31014-s2.pdf]

The CD4$^+$ T cell methylome contributes to a distinct CD4$^+$ T cell transcriptional signature in *Mycobacterium bovis*-infected cattle

Rachael Doherty[1, 2], Ronan Whiston[1, 2], Paul Cormican[1], Emma K. Finlay[1], Christine Couldrey[3], Colm Brady[4], Cliona O'Farrelly[2] and Kieran G. Meade[1].

Supplementary Tables

Table s1 RNA-seq mapping statistics

Table s2 List of differentially expressed genes (FDR <0.1), including their corresponding log_2FC, log_2CPM and FDR corrected p-values.

Table s3 RRBS mapping statistics

Table s4 List of differentially methylated sites/regions (change >25%, q <0.01)

Table s5 Primers used for qRT-PCR amplification of *TGFB1* in peripheral blood leukocytes of *M. bovis* infected cattle and for qMethyl analysis of IFNG gene promoter methylation

Table S1	FastQC	Bowtie2 & Tophat		FlagStat	HtSeq-Count	
Animal ID	Starting Reads	Aligned Reads	% Aligned	% Properly Paired	No Feature	Ambiguous
Control 1	80,593,632	50,798,166	63.03%	62.95%	8,689,384	715,146
Control 2	87,930,604	58,403,507	66.42%	68.98%	8,134,612	770,941
Control 3	73,040,780	45,445,973	62.22%	59.40%	8,317,263	623,031
Control 4	78,402,918	50,318,993	64.18%	64.27%	8,577,859	663,179
Control 5	65,321,920	46,032,357	70.47%	65.30%	5,145,101	650,186
Infected 1	68,876,202	44,094,545	64.02%	66.29%	7,477,892	574,942
Infected 2	61,438,858	36,703,574	59.74%	39.32%	7,616,292	628,083
Infected 3	64,823,940	39,970,441	61.66%	54.94%	7,428,734	630,633
Infected 4	76,297,562	47,266,340	61.95%	62.27%	8,745,286	626,272
Infected 5	74,122,330	41,678,986	56.23%	64.67%	10,605,520	516,536

CD4+ T lymphocytes were isolated from the PBMC fraction and high quality DNA was extracted and purified for all animals. RRBS libraries were prepared commercially and the 10 libraries were pooled before final quantification using the Bioanalyzer. Libraries were confirmed to contain adapter ligated fragments of the correct length, ranging from 150-460 bp. Libraries were then commercially sequenced on an Illumina HiSeq 2000.

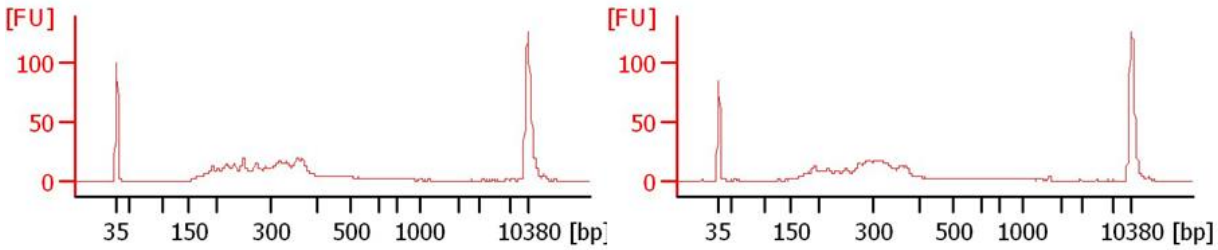

Bioanalyzer trace of pooled RRBS libraries

Pooled RRBS libraries generated from bovine genomic DNA were confirmed to contain adapter ligated fragments between 150-460 bp in length.

Sequence data analysis

RRBS sequence data was generated using 50 base pair paired end reads. An initial assessment of read quality was carried out using FastQC software, which analyses the sequence data across eleven different quality control modules. The quality of the sequence data was assessed using Phred quality scores which are assigned to each nucleotide. The average Phred score for this data was above 30 indicating very high quality sequence data for downstream analysis. High quality sequence reads were mapped to the bovine genome (BTau7) using the software tool Bowtie in conjunction with the mapping software Bowtie. Percentage mapping efficiencies ranged from 25.5% to 44.9%. Mapping statistics for all samples are displayed in Table s2, along with the overall methylation percentage for each library.

Table s2 Summary of all reads pre and post mapping to the bovine genome

The starting number of reads, those retained after mapping to the genome and the average CpG methylation percentage across the genome is shown.

Sample ID	Bisulfite conversion	Total reads	Mapped reads	Mapping %	Methylation %
Control 1	99.15%	33,270,443	8,474,462	25.5%	62.5%
Control 2	99.27%	26,910,772	9,705,924	36.1%	65.8%
Control 3	99.36%	35,155,304	15,778,818	44.9%	63.6%
Control 4	99.40%	35,886,917	13,743,205	38.3%	67.8%
Control 5	99.33%	27,033,701	11,976,534	44.3%	63.2%
Infected 1	99.34%	31,231,275	11,589,711	37.1%	67.4%
Infected 2	98.02%	64,913,395	18,166,754	28.0%	68.7%
Infected 3	99.21%	23,454,957	9,140,528	39.0%	65.8%
Infected 4	99.29%	33,231,205	11,047,714	33.2%	68.0%
Infected 5	99.21%	39,362,319	14,952,405	38.0%	66.2%

Table S5

Primers for qMethyl PCR amplification of the IFNG promoter

Primers were designed to cover > 3 recognition sites for methylation sensitive restriction enzymes and to amplify a region of 303 bp in length.

Gene ID	Primer sequence	Product size (bp)
IFNG	GCATCTCTGTCTATCTGTCACCA	303 (-137/-166)
	CCTGGCCATAAGAACCAGAA	

Primers for qRT-PCR of TGFB1 and GAPDH

Gene ID	Primer sequence (F)	Primer sequence (R)
TGFB1	TGCTTCAGCTCCACAGAAAAGA	AGGCAGAAATTGGCGTGGT
GAPDH	CTCCCAACGTGTCTGTTGTG	TGAGCTTGACAAAGTGGTCG
